# Subthreshold opioid use disorder prevention (STOP) trial: a cluster randomized clinical trial: study design and methods

**DOI:** 10.1186/s13722-023-00424-8

**Published:** 2023-11-18

**Authors:** Jane M. Liebschutz, Geetha A. Subramaniam, Rebecca Stone, Noa Appleton, Lillian Gelberg, Travis I. Lovejoy, Amanda M. Bunting, Charles M. Cleland, Karen E. Lasser, Donna Beers, Catherine Abrams, Jennifer McCormack, Gail E. Potter, Ashley Case, Leslie Revoredo, Eve M. Jelstrom, Margaret M. Kline, Li-Tzy Wu, Jennifer McNeely

**Affiliations:** 1 Division of General Internal Medicine, Center for Research On Health Care, University of Pittsburgh, 200 Lothrop Street, Suite 933W, Pittsburgh, PA 15213 USA; 2 National Institute On Drug Abuse, Bethesda, MD USA; 3 The Emmes Company, LLC, Rockville, MD USA; 4 Department of Population Health, NYU Grossman School of Medicine, New York, NY USA; 5 David Geffen School of Medicine at UCLA, UCLA Fielding School of Public Health, Los Angeles, CA USA; 6 Department of Psychiatry, Oregon Health & Science University, Portland, OR USA; 7 Section of General Internal Medicine, Boston Medical Center, Boston University Chobanian & Avedisian School of Medicine, Boston, MA USA; 8 School of Public Health, Boston University, Boston, MA USA; 9 Emory University School of Medicine, Atlanta, GA USA; 10 Biostatistics Research Branch, NIH/NIAID, Rockville, MD USA; 11 Department of Psychiatry and Behavioral Sciences, Duke University School of Medicine, Durham, NC USA

**Keywords:** Risky Opioid Use, Primary Care, Collaborative Care, Cluster-Randomized controlled trial, Substance use disorder, Prevention, Opioid use disorder

## Abstract

**Background:**

Preventing progression to moderate or severe opioid use disorder (OUD) among people who exhibit risky opioid use behavior that does not meet criteria for treatment with opioid agonists or antagonists (subthreshold OUD) is poorly understood. The Subthreshold Opioid Use Disorder Prevention (STOP) Trial is designed to study the efficacy of a collaborative care intervention to reduce risky opioid use and to prevent progression to moderate or severe OUD in adult primary care patients with subthreshold OUD.

**Methods:**

The STOP trial is a cluster randomized controlled trial, randomized at the PCP level, conducted in 5 distinct geographic sites. STOP tests the efficacy of the STOP intervention in comparison to enhanced usual care (EUC) in adult primary care patients with risky opioid use that does not meet criteria for moderate-severe OUD. The STOP intervention consists of (1) a practice-embedded nurse care manager (NCM) who provides patient participant education and supports primary care providers (PCPs) in engaging and monitoring patient-participants; (2) brief advice, delivered to patient participants by their PCP and/or prerecorded video message, about health risks of opioid misuse; and (3) up to 6 sessions of telephone health coaching to motivate and support behavior change. EUC consists of primary care treatment as usual, plus printed overdose prevention educational materials and an educational video on cancer screening. The primary outcome measure is self-reported number of days of risky (illicit or nonmedical) opioid use over 180 days, assessed monthly via text message using items from the Addiction Severity Index and the Current Opioid Misuse Measure. Secondary outcomes assess other substance use, mental health, quality of life, and healthcare utilization as well as PCP prescribing and monitoring behaviors. A mixed effects negative binomial model with a log link will be fit to estimate the difference in means between treatment and control groups using an intent-to-treat population.

**Discussion:**

Given a growing interest in interventions for the management of patients with risky opioid use, and the need for primary care-based interventions, this study potentially offers a blueprint for a feasible and effective approach to improving outcomes in this population.

*Trial Registration*: Clinicaltrials.gov, identifier NCT04218201, January 6, 2020.

**Supplementary Information:**

The online version contains supplementary material available at 10.1186/s13722-023-00424-8.

## Introduction

Responses to the opioid crisis have focused on decreasing opioid prescribing, reducing fatal overdoses, and increasing access to medications for opioid use disorder (OUD). Little attention has been paid to preventing the progression to moderate or severe OUD among people who exhibit risky opioid use behavior, which includes nonmedical use of prescribed opioids or any use of illicit opioids. Subthreshold OUD consists of risky opioid use (e.g. taking illicit opioid or nonmedical use of prescription opioid) that does not meet the threshold for treatment with opioid agonists or antagonists (e.g. moderate or severe OUD as defined by DSM-V criteria of 4 or more criteria) [1]. Patients with subthreshold OUD may experience few symptoms or consequences of their opioid use and rarely seek addiction treatment services [2, 3], but they are frequently encountered in primary care settings where they receive routine medical care and, in some cases, prescription opioids for pain management. Although prevalence in primary care settings varies based on the patient population, 4.7% of all adults are estimated to have risky opioid use, [4], and prevalence is much higher (21–29%) among those receiving prescribed opioids [5–7]. Primary care clinics are optimally positioned to identify and provide early intervention for individuals with risky opioid use because they are the largest prescribers of opioid analgesics [8], and they are often the only point of health care contact for individuals with illicit opioid use that has not yet progressed to OUD. Yet there has been almost no research on interventions to reduce the potential harms of opioid use or halt the progression to OUD among primary care patients with subthreshold opioid use [9].

Most prior approaches to reducing subthreshold drug use in general medical settings have relied on single-contact brief interventions [10–13], delivered using ‘screening, brief intervention, and referral to treatment (SBIRT)’ models. In randomized clinical trials, these single-contact brief interventions have had mixed results for reducing drug use, with most studies showing no difference compared to no intervention. Those that were positive showed small reductions and were limited by having short-term follow-up [13–17]. A pilot study by Bohnert and colleagues in an Emergency Department (ED) setting utilized a 30-min therapist delivered motivational interview and found decreased self-reported opioid use and misuse in individuals at risk for overdose [15]. With a slightly more intensive but still limited intervention, Gelberg and colleagues conducted the Quit Using Drugs Intervention Trial (QUIT) which tested brief advice by the primary care provider (PCP) and video doctor, with 2 sessions of motivational counseling by a telephone health coach in primary care clinics for individuals with any moderate-risk illicit drug use. In two trials, the QUIT intervention reduced self-reported drug use by 33%-44% compared to usual care [16, 17].

Collaborative care is an established approach for improving mental health conditions in primary care patients, [18–20] and could potentially be an effective approach for subthreshold opioid use. Formal collaborative care models include population management, patient-centered team-based care, evidence based treatment, measurement based care and accountability [18–20]. Collaborative care is grounded in the Chronic Care Model (CCM) for chronic disease management, [21–24] which seeks to improve patient outcomes through integrative systems changes, including organizational support, clinical information systems, delivery system redesign, decision support, patient self-management support, and community resources. Collaborative care teams typically consist of a PCP working with a care manager who maintains a clinical registry to proactively track and manage care delivery [24]. Prior interventions for substance use have included elements of formal collaborative care, such as interdisciplinary team-based care, as exemplified in the Massachusetts nurse-care model [25]. TOPCARE (Transforming Opioid Prescribing in Primary Care) and TEACH (Teaching Effective Analgesia in Clinics for HIV) were cluster randomized trials that employed a nurse care manager using population management tools, academic detailing, and web-based decision support. While these trials improved PCP delivery of guideline concordant opioid prescribing practices for patients receiving chronic opioids for pain in primary care and HIV care, respectively,[26–28] they did not examine patient outcomes.

The Sub-Threshold Opioid Use Disorder Prevention (STOP) Trial is designed to study the efficacy of elements of a collaborative care intervention to reduce risky opioid use and to prevent progression to moderate or severe OUD in adult primary care patients with risky opioid use. The STOP intervention augments previously tested care models by combining PCP brief advice and telephone health coaching along with a nurse care manager working with the PCP. The STOP intervention thus combines patient-facing and provider-facing components. The purpose of this report is to describe the STOP trial protocol.

## Methods

### Objectives and hypotheses

The primary objective (Aim 1) is to determine the efficacy of the STOP collaborative care intervention, in comparison to enhanced usual care (EUC), for reducing risky opioid use in adult primary care patients. Risky opioid use is defined as nonmedical use of prescribed opioids (e.g. more than prescribed or non-prescribed opioids) or any use of illicit opioids. We hypothesize that patient participants with PCPs assigned to the STOP intervention, relative to patient participants with PCPs assigned to EUC, will have fewer days of risky opioid use, measured at 6 months post-baseline (primary outcome), and at 3-, 9-, and 12-months post-baseline (secondary outcomes).

Secondary objectives capture patient participant (Aim 2) and provider participant-level outcomes (Aim 3). We hypothesize that patient participants with PCPs assigned to the STOP intervention, in comparison to those with providers assigned to EUC, will have improved measures of other substance use, quality of life, mental health symptoms and no worsening of pain severity. For PCPs in the study (provider participants), we hypothesize that providers assigned to the STOP intervention, in comparison to those assigned to EUC, will adopt improved treatment practices that include safe prescribing of controlled substance and increased monitoring for patients with subthreshold OUD. Specific secondary outcomes are detailed below and in the Additional file 1.

### Overview of trial design

The STOP trial is a cluster randomized controlled trial that tests the efficacy of the STOP intervention to reduce opioid use and overdose risk, and to prevent progression of OUD in adult patients with risky opioid use over the course of 12 months, as compared to EUC. It is being conducted within the NIDA Clinical Trials Network, as part of the NIH Helping to End Addiction Long-term (HEAL) Initiative.[29] Five geographically distinct study sites, each having one or more participating primary care clinics, are participating. PCPs are randomized, stratified by site, in a 1:1 ratio to either the STOP intervention or control EUC condition. In the case of one site where multiple PCPs practice as a team (i.e., share patient care among multiple PCPs in the team), the team of PCPs together counts as a single cluster for randomization. Patients who are eligible and enroll receive the intervention according to the assignment of their PCP. EUC consists of primary care treatment as usual, plus receipt of printed educational materials addressing opioid-related overdose prevention and an educational video on cancer screening.

The STOP intervention is a collaborative care model consisting of (1) a practice-embedded nurse care manager (NCM) who provides patient participant education and supports the PCP in engaging and monitoring patients who have risky opioid use; (2) brief advice delivered to patient participants by their PCP and/or prerecorded video message from a study investigator (LG) about health risks of opioid misuse; and (3) at least 2 sessions, and up to 6 sessions, of telephone health coaching to motivate and support behavior change amongst patient participants.

**Fig. 1:**
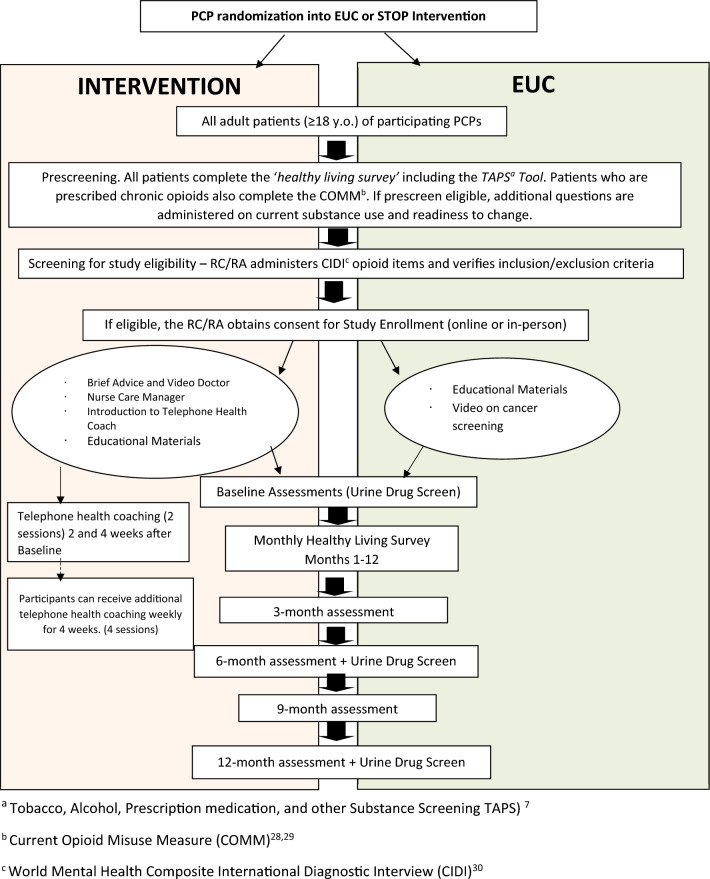
Basic Study Schema

Prior to enrollment, patients are informed that their PCP is participating in a “healthy living study” and are blinded to both the study condition of their PCP and the focus of the study (risky opioid use). After enrollment it is possible that they may be able to deduce their PCP’s assignment, but this information will not be volunteered by clinical or research staff. To assess the success of the blind, patient participants will be asked at their last study visit what they considered to be the goal of the research. Patient participants are assessed through multiple modalities, including monthly brief surveys sent via text message and email, structured questionnaires delivered over the phone and via a web-based portal at baseline and quarterly for 12 months, and urine drug screens at baseline, 6 and 12 months. PCP prescribing and monitoring behaviors are assessed from the electronic health record (EHR) as well as self-administered questionnaires regarding attitudes and behaviors on opioid prescribing. An independent commercial Institutional Review Board (IRB) approved the study, and all sites ceded to this central IRB Fig. 1.

### Conceptual model

**Fig. 2:**
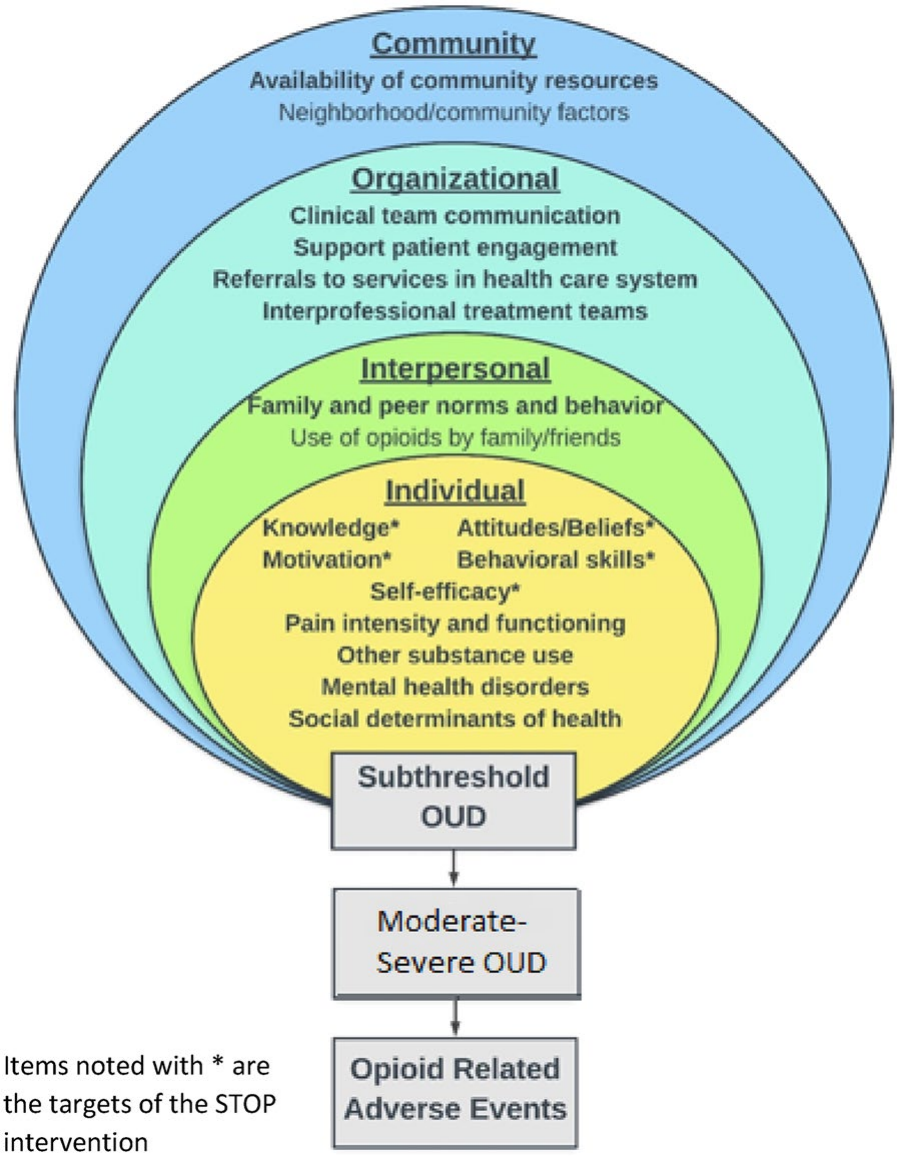
Social Ecological Framework of Risky Opioid Use

The STOP collaborative care intervention integrates patient- and provider-facing interventions and is informed by Social Ecological Frameworks for health promotion [30], and the opioid use literature. As depicted in Fig. 2, subthreshold OUD is influenced by multiple factors, including (1) patient-level individual factors such as social determinants of health and mental health disorders; (2) interpersonal factors such as use of opioids by family/friends; (3) clinic organizational factors such as utilization of clinical information systems to enhance communication and efficiency; and (4) community factors such as availability of community resources and other neighborhood characteristics. While STOP does not directly intervene on all the factors that impact a patient’s risky opioid use, it is designed to address each level of the social ecological framework. Additional file 1: Appendix Table S1 maps the STOP intervention components to the Social Ecological Framework’s four levels of influence. Addressing each of these multiple systems of influence is expected to support individuals with subthreshold OUD to reduce their risky opioid use behaviors, which can in turn reduce the risk of escalation to moderate-severe OUD and opioid-related events, such as overdose and death.

### Trial registration

Prior to recruitment, the trial was registered at clinicaltrials.gov, identifier NCT04218201.

### Site selection

The study team distributed a call for applications to be a clinical site for the trial to the NIDA Clinical Trials Network, which comprises 16 geographically distinct nodes, each of which is affiliated with one or more large academic centers and multiple clinical sites. Eligible sites were expected to have approximately 7–30 PCPs who would be eligible to participate, capacity to provide referrals to opioid treatment program(s), an electronic health record, a clinical champion willing to work with the study team to facilitate study clinical activities, and space for a nurse care manager. Participating sites could not have an existing collaborative care model dedicated to managing patients receiving opioid prescriptions. Although not required, additional desirable characteristics included location in a region with higher-than-average prevalence of opioid use, capacity to provide in-office buprenorphine treatment, ethnic/racial diversity, and space in or near the clinic for research activities.

The study team conducted site visits with potential clinics from June to September 2019 and the sites were finalized in November 2019.

### Clinical champion

Each site has a Clinical Champion, (4 sites had one, 1 site had two) who is a physician, or in one case a clinical psychologist, actively practicing in the study clinics. The role of the Clinical Champion is to help recruit PCPs and serve as a resource for the nurse care manager as well as PCPs randomized to the STOP intervention.

### PCP eligibility

Eligible PCPs are licensed medical professionals (MD, DO, NP, PA) who have a total weekly patient volume of approximately 40 or more adult patients during a typical week and provide care to approximately 4 or more adult patients receiving chronic opioid therapy and/or with risky opioid use. Chronic opioid therapy is defined as having at least three opioid prescriptions, at least 21 days apart, in the past six months, with EHR documentation of active opioid prescription within 60 days prior to screening. The PCPs must be willing to be randomized to either study condition. They are excluded if they are medical residents or have definite plans to resign from the clinic in the subsequent 24 months or change their schedule in the next 24 months such that they would no longer meet the inclusion criteria for patient volume, per PCP self-report.

### PCP recruitment

The Site PI and/or Clinical Champion work with clinic staff to identify potentially eligible PCPs who they approach to assess their interest in participating. Interested PCPs complete a screening survey to assess eligibility and if eligible, research staff conduct informed consent and the PCP completes a baseline survey (see below, study measures). The Clinical Champion delivers orientation to the PCPs randomized to the STOP intervention (see below, training).

### Patient eligibility

Patient eligibility criteria include (1) PCP is enrolled in the study. (2) Age ≥ 18 at time of prescreening. (3) Proficient in written and spoken English, as determined by self-report and research staff. (4) Access to phone that can receive text messages. (5) Access to a phone that could receive text messages and to the internet (via smartphone, tablet, computer). (6) Sufficient contact information (minimum of 1 reliable locator). (7) Ability to provide informed consent. (8) Risky opioid use, defined as a Tobacco, Alcohol, Prescription medication, and other Substance Screening TAPS [7] score ≥ 1 for illicit opioid (heroin or fentanyl) use and/or nonmedical prescription opioid use, and/or a positive response (> Never) to Current Opioid Misuse Measure (COMM)[32, 33] items indicating taking more opioid medication than prescribed or opioid medications that belonged to someone else, on any of the following three items: (Item 9) In the PAST 30 DAYS, how often have you needed to take pain medications belonging to someone else?; (Item 14) In the PAST 30 DAYS, how often have you had to take more of your medication than prescribed?; (Item 15) In the PAST 30 DAYS, how often have you borrowed pain medication from someone else?

Exclusion criteria include: (1) Moderate-severe opioid use defined as meeting 4 or more DSM-5 criteria for OUD at screening, as assessed by research staff administering the World Mental Health Composite International Diagnostic Interview (CIDI) modified for DSM-5 criteria (removing legal problems and adding craving) [34]. (2) Receiving medication for OUD or engaged in an opioid treatment program in the past 30 days from screening date. (3) Receiving opioids for end-of-life care. (4) Pregnancy, as determined by self-report. (5) Currently in jail, prison, other overnight facility as required by court of law, or have pending legal action. (6) Definite plans to leave the area or clinic practice within 12 months. (7) Other factors that may cause harm or increase risk to participant.

### Patient recruitment

Research staff (research coordinator (RC) or research assistant (RA)) prescreen patients of participating PCPs for eligibility. Although the study was initially conceived as primarily conducting prescreening in clinic waiting rooms, with the COVID pandemic related restrictions, procedures were adapted to offer prescreening in multiple ways, with a reliance on remote methods. Procedures include mailing letters to patients, sending messages to patients through email and/or patient portals, texting and calling patients, posting flyers in clinical areas, and clinical staff referrals. When possible, research staff may also conduct in-person waiting room prescreening.

### Patient prescreening for eligibility

The prescreen is conducted via a self-administered web-based “Healthy Living Survey”, which includes questions about general health behavior (exercise, diet) as well as substance use. The purpose of including general health questions is to partially mask the opioid focus of the study for potential participants. For all patients, the prescreening survey includes the TAPS Tool [7]. For patients who report receiving an opioid medication prescription in the previous 6 months, the prescreening tool additionally includes the COMM [32, 33].

To protect anonymity, prescreening forms do not include identifying information for those who are ineligible. If a patient prescreens eligible, they are asked to create a unique ‘study ID’ and leave their contact information for the research staff to reach them. Patients who do not qualify for the study have no further study interaction.

### Screening and enrollment

The RC or RA contacts individuals who have prescreened eligible to conduct further ascertainment of eligibility and to administer informed consent. To conduct the eligibility screen, the RC/RA administers the CIDI opioid items and completes a checklist of inclusion and exclusion criteria. The RC/RA obtains written informed consent from eligible and interested individuals.

Individuals who are ineligible on screening due to moderate to severe OUD or receipt of MOUD will receive from the RC/RA information about overdose prevention, a list of community and/or clinic resources for OUD treatment, contact information for a social worker or behavioral health staff member at the clinic, an ineligible patient letter patients can provide to their PCP (for moderate to severe OUD patients only); and the RC/RA will suggest that patients speak with their PCP. They are not informed why they were ineligible.

### Study treatment arms

The two study arms include Enhanced Usual Care (EUC) and the STOP intervention. The interventions do not interfere with usual care received by patient participants and could include referral to treatment for OUD if patient participants in either arm of the study should develop moderate-severe OUD during the study. Of note, patients in the study could be receiving prescription opioids from any provider, including the PCP but also non-PCP prescribers.

### Enhanced usual care (EUC)

In the EUC arm, PCPs conduct primary care as usual, without the support of the NCM or health coach, and they are not made aware that their patient is enrolled in the trial. The enhancement occurs at the time of the initial baseline visit when patient participants view a short video and receive written materials on cancer screening and educational materials on opioid-related overdose prevention, including how to obtain a naloxone kit. The justification for the EUC is that current practice in primary care generally does not include routine opioid safety measures, including overdose prevention information or naloxone acquisition. The use of the cancer screening video and handouts are to enhance blinding of the study purpose and serve as an attention control.

### STOP intervention

The STOP intervention consists of three key components: Brief advice delivered by the PCP and/or a brief video doctor on opioid use, two to six sessions of telephone health coaching, and 12 months of nurse care management. Each component was included in prior clinical trials conducted in primary care settings that were effective in decreasing drug use [16, 17] or for increasing guideline-adherent opioid prescribing [27, 28, 35]. The STOP intervention combines them. Intervention participants also receive educational materials on opioid-related overdose prevention given to EUC participants. See Table 1.

**Table 1:** Summary of Intervention Provider Roles and Timing of Patient Contacts

Team member	PCP	NCM	Telephone Health Coach
*Role and responsibilities*	• Brief advice • Discusses opioid use in context of overall health	• Health education • Risk reduction counseling • Overdose prevention and naloxone provision • Self-management skills • Education on non-pharmacologic pain management • Referrals and support for pain, mental health, SUD treatment, other services • Healthcare and resource navigation • Supports engagement in primary care • Uses motivational enhancement strategies to engage patients in SUD treatment when indicated • Monitoring opioid prescribing and risks, when applicable	2 initial coaching sessions • Offers support • Evidence-based counseling (MI) to enhance motivation for substance use behavior change • Risk reduction counseling • Suggests strategies for overcoming barriers to behavior change • Encourages engagement w/ primary care and behavioral health providers and study nurse care manager in the clinic • Encourages utilization of community and/or on-line resources to support behavior change 4 enhanced coaching sessions: • Evidence-based CBT focused on substance use behavior change • Additional CBT to address other patient concerns (Pain, Depression, Anxiety)
*Timing of patient contacts*	1.Baseline visit or within 10 business days 2.Readdress in follow-up primary care visits	1.Baseline visit 2.12 months of ongoing monitoring and engagement in conjunction with PCP. Meets on an approximately monthly basis for patients prescribed chronic opioids and as needed for all patients	1.Initial health coaching: Week 2 and Week 4 (approximately) following baseline visit 2.Enhanced coaching: For individuals who may benefit from further intervention: Weeks 7,8,9,10 (approximately)
*Location*	Clinic, video visit, or telephone visit	Clinic (contacts are in-person ± by telephone)	Telephone and/or video

#### I. Brief Advice

After a patient participant enrolls, the RA/RC informs the patient’s PCP and provides the PCP with a printed ‘Summary to the Clinician’ that explains the patient participant’s prescreening results (TAPS and, if applicable, COMM) and a script that guides the PCP in providing brief advice on reducing risky opioid use. The scripts are adapted from those used in the QUIT trial [16, 17] and are available in two versions—one for patients misusing illicit opioids and the other for patients misusing prescribed opioids. See Additional file for copies of scripts.

For patient participants with an upcoming scheduled primary care visit at the time of enrollment, PCPs are asked to give brief advice at the time of the visit. If the participant does not have a scheduled primary care visit, the PCPs are asked to call the patient to deliver the brief advice within 10 business days. Afterwards, PCPs fill out an intervention checklist about the advice given (or not given).

All patient participants are asked by research staff to watch a ‘Video Doctor’ recording during the baseline study visit to supplement any brief advice that is delivered by the PCP, as utilized in the QUIT trials [16, 17]. The 5-min video features a 3-min clip from a study investigator (LG) delivering the same brief advice script that is given to the PCP, as well as videos of the health coaches and local nurse care manager introducing themselves and their roles.

#### II. Telephone health coach

The Telephone Health Coaches (hereafter referred to as Coaches) deliver a series of individual health coaching sessions. The coaches are not affiliated with the clinics but provide a national phone bank available to patients of any of the study clinics. This allows patients to discuss clinic related barriers they face and to discuss issues that they do not want recorded in their EHR. The Coaches utilize elements of Motivational Interviewing (MI) and Cognitive Behavioral Therapy (CBT) to improve patient participant motivation and self-efficacy to change health behavior and behavioral outcomes [36–38]. Health coaches may be social workers, counselors, or individuals with other professional training that qualifies them to deliver the STOP counseling intervention.

All patient participants in the STOP arm are offered the two initial health coaching sessions with or without video, based on patient participant preference, at approximately 2- and 4-weeks post-baseline. See Table 1 for content of coaching sessions.

Enhanced coaching (Step 2):

Patient participants who may benefit from additional coaching following the Step 1 sessions are offered up to 4 additional sessions with the same Coach. These sessions ideally start within two weeks of the patient participant’s referral into Step 2 and occur once a week for four consecutive weeks. Enhanced coaching may be recommended by the NCM or PCP, or offered at the discretion of the Coach, based on patient preference as well as clinical criteria which may include ongoing or escalating nonmedical or illicit opioid use, overdose risk behavior, symptoms of OUD, overdose episodes, and/or consequences of opioid and other substance use. Enhanced coaching is offered by the Coach at the time of the second Step 1 session, and patients are free to decline. (Table 1).

#### III. Nurse care manager

The Nurse Care Manager (NCM) works closely with the intervention PCP to assess, educate, and manage patient participants with risky opioid use. Details of the NCM intervention are outlined in Table 1. Patient participants are asked to schedule an initial visit with the NCM as soon as possible following study enrollment. The NCM continues working with patients in the STOP condition throughout their 12 months of study participation. Following the initial visit with the NCM, the frequency of visits depends on patient participant needs. Throughout the health coaching period, the Coaches and NCMs meet weekly to discuss their patients and strategize how to effectively work with the entire care team to support the patient.

#### Training

Each of the professionals involved in the intervention receives specific training prior to patient recruitment.

The Clinical Champions receive training conducted by two primary care physicians with expertise substance use (LG and Lead Investigator JM). The primary goal of the training is to prepare Champions to educate and provide guidance to their PCP colleagues. The training orients them to the study and covers the Summary to the Clinician, delivery of brief advice, management and monitoring of patients receiving prescription opioids, identifying, and assessing OUD, treatment of OUD, and overdose prevention.

PCP participants randomized to the intervention receive a 20–30-min training delivered by the site Clinical Champion. This training includes practicing delivery of brief advice, understanding the Summary to the Clinician, documentation on the intervention checklist, and collaboration with the NCM. PCP participants in the EUC condition only receive an introduction to the study during the Clinical Champion’s initial presentation to the clinic.

Health Coaches undergo initial training and then participate in a weekly virtual Learning Community (as in the QUIT trials [16, 17]) for ongoing technical support, mutual problem solving, supervision, and to maintain fidelity of the intervention. Some of the training sessions occur jointly and some are role specific. Study trainers include a PhD clinical psychologist (TL) and a physician expert in health coaching for risky substance use (LG). Training is delivered virtually over a 6-day period and is focused on the content and procedures involved in behavior change coaching, with special focus on building competence in motivational interviewing and cognitive behavioral therapy techniques. Coaches and NCMs learn techniques to refer patient participants to local resources as well as to assess suicidality. After formal training, the health coaches conduct mock sessions, which are audio recorded and coded for fidelity by the trainers.

NCMs undergo 10 h of didactic training over the course of 5 days, and an additional 4–8 h of interactive workshop training prior to the start of their participation in the study. These sessions are led by two experienced nurse care managers and the study Lead Investigator (DB, CA, JL), all of whom participated in prior trials using NCM for opioid prescribing. Throughout the study, these trainers facilitate a weekly hour-long group meeting of the NCMs to offer technical support.

### PCP participant compensation

Provider participants receive compensation in the form of debit cards or gift cards for completing surveys ($25 per survey, total of $50).

### Patient participant compensation

Patient participants receive compensation in the form of debit cards or gift cards for completing study activities including surveys, urine drug screening, and updating contact information (total possible $670 for full participation). Intervention patients also get $3 per telephone health coaching to offset the cost of data usage on mobile phones (maximum $18) but are not otherwise compensated for participation in any aspect of the intervention. A subsample of intervention patients who undergo qualitative interviews to assess the intervention experience receive a further $50.

### Study assessments

#### Sources of data

Assessments and times of their administration are listed in Table 2. Outcome data are collected from patient participants via self-administered surveys, RA/RC-administered surveys, and urine toxicology tests. Self-administered assessments were selected whenever possible, to increase feasibility and limit social desirability bias. Data from PCP participants are collected via self-administered surveys, and through audit of the electronic health record. In addition, a research staff member conducts qualitative interviews by telephone with a convenience sample of patient participants between three and six months of study participation, with distribution across sites.

**Table 2:** Aims, Outcomes and Measures

Outcome	Instrument	Time of administration
Aim 1: Determine efficacy of STOP as compared to EUC for reducing risky opioid use		
Risky Opioid Use	Healthy Living Survey	Baseline, monthly
	Timeline Follow-Back [76]	3 m, 6 m
	Urine Toxicology Screen	Baseline, 6 m, 12 m
Aim 2: Examine the impact of STOP on important patient-level outcomes		
Substance Use		
Binge alcohol use, benzodiazepine use, stimulant use, other drug use	Healthy Living Survey	Baseline, monthly
	Urine Toxicology Screen	Baseline, 6 m, 12 m
Tobacco	E-cigarette and vaping	Baseline, 6 m, 12 m
Marijuana use	Healthy Living Survey	Baseline, monthly
	Marijuana Use assessment [77]	Baseline, 12 m
Prescription opioid misuse behaviors among individuals with prescribed opioids	Current Opioid Misuse Measure (COMM) [32]	Baseline, 6 m, 12 m
Incidence of Moderate-Severe OUD	CIDI [34]	Baseline, 6 m, 12 m
Overdose risk behavior	Non-fatal overdose questionnaire [47]	Baseline, 6 m, 12 m
Other outcomes		
Alcohol and drug use disorder	PDSQ [42, 43, 78]	Baseline, 6 m, 12 m
Pain symptoms and functioning	BPI – short form [79]	Baseline, 3 m, 6 m, 9 m, 12 m
Depression and Suicide	PHQ-8 [80], PSS [81, 82]	Baseline, 6 m, 12 m
Anxiety	PROMIS anxiety short form [41]	Baseline, 6 m, 12 m
Sleep Quality	PROMIS sleep 4a [83]	Baseline, 6 m, 12 m
Health Related Quality of Life	SF-12 measure [84]	Baseline, 6 m, 12 m
Health Care Utilization	Patient reported ED visits and hospitalizations	Baseline, 6 m, 12 m
Aim 3: Characterize the impact of STOP on primary care provider behaviors		
Rates of opioid prescribing, including high dose	Chart Abstraction	12 m
Rates of Benzodiazepine prescribing	Chart Abstraction	12 m
Receipt of naloxone kit^a^	Chart Abstraction	12 m
	Nurse Care Manager checklist	Cumulative 12 months
Monitoring for patients prescribed opioids	Chart Abstraction	12 m
Other outcomes		
Organizational context in readiness to change	ORCA Context Items [85]	Baseline
Experience with assessment and treatment of pain, COT management	Modified TOPCARE survey [26]	Baseline, 12 m
Substance use and misuse in patients: knowledge and attitudes	Modified ASIP survey [86]	Baseline, 12 m
Experience with management of risky opioid use, OUD, overdose prevention, X-waiver	Modified TOPCARE survey [26]	Baseline, 12 m
Feedback on intervention (intervention PCPs only)	QUIT [16, 17]	12 m

BPI = Brief Pain Inventory

CIDI = Composite International Diagnostic Interview

PDSQ = Psychiatric Diagnostic Screening Questionnaire

PHQ = Patient Health Questionnaire

PROMIS = Patient-Reported Outcomes Measurement Information System

PSS = Patient Safety Screener (PSS)

TLFB = Timeline Follow Back

^a^Because each site has different regulations on naloxone distribution, this would encompass either directly supplying naloxone or referring patient to place to obtain naloxone

Patient participants are administered the “Healthy Living Survey” at baseline and monthly via email/text with a computerized link to questions quantifying use of opioids and non-opioid substances, and prescription opioid misuse. Questions on diet, exercise, and stress are included to blind the study participants as to the study focus.

Primary Outcome: Days of Risky Opioid Use.

The primary outcome measure is self-reported number of days of risky opioid use in the past 180 days, assessed at 6 months after the baseline visit using single item questions based on the Addiction Severity Index [39, 40] and the COMM [32, 33] via the “Healthy Living Survey” (See Text box below). Participants are asked to specify the number of days of illicit opioid use and of nonmedical use of prescribed opioids in the past 30 days (range is 0–30 days). Illicit opioid use includes use of heroin or fentanyl. Nonmedical opioid use includes using prescribed opioids more frequently or at higher doses than instructed on the prescription (e.g., taking 2 tablets when the prescription indicates a dose of 1 tablet), or taking pharmaceutical opioids that were not prescribed to them. The extent of overlap between these different types of risky opioid use is also measured, when applicable. The measure is calculated as the sum of all days of use reported on the assessments of past 30-day drug use for the first 6 months (i.e., the sum of days of use from the measures collected on day 30, day 60, day 90, day 120, day 150, and day 180 minus the overlap). For example, if a participant reports nonmedical opioid use on 10 days, illicit opioid use on 12 days, and there were 4 days on which they used both nonmedical and illicit opioids, then the total number of days of risky use are calculated as 10 + 12–4 = 18.Days of risky opioid use are assessed at the prescreen eligibility survey and every 30 days for the 12 months of study participation. The assessment is self-administered using a computerized form delivered using a text message or email 
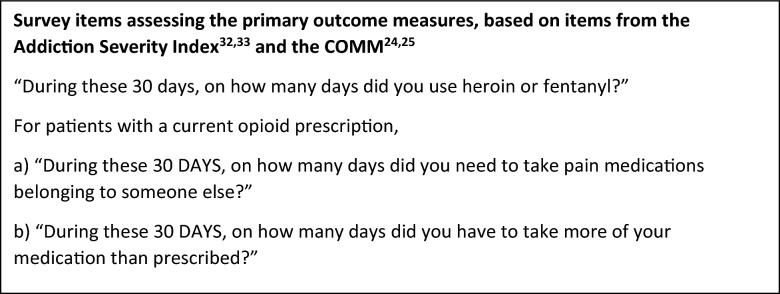
link to a monthly Healthy Living survey.

### Secondary outcomes

Substance Use: The Healthy Living Survey includes other items on opioid and other substance use for secondary analyses. Substances queried using additional questions based on the Addiction Severity Index [39, 40] include the number of alcohol heavy drinking days (4 + drinks/day for women, 5 + drinks/day for men), and days of nonmedical use of benzodiazepines or prescription stimulants,, illicit stimulant (cocaine or methamphetamine), marijuana, and other drugs. Additional items adapted from the COMM [32, 33] include: “During the PAST 30 DAYS, on how many days did you borrow pain medication from someone else?” and “During the PAST 30 DAYS, on how many days did you use your pain medication for symptoms other than for pain (e.g., to help you sleep, improve your mood, or relieve stress)?”. Days of non-opioid substance use is measured using the same approach as days of risky opioid use, with one question asking about number of days of use of each substance type in the past 30 days, but without questions about overlapping days of use.

Other sources of data on substance use include qualitative urine drug screens to verify self-reported drug use at baseline, 6, and 12 months. The urine drug screen measures opioids, oxycodone, fentanyl, barbiturates, benzodiazepines, cocaine, amphetamine, methamphetamine, marijuana, methadone, buprenorphine, phencyclidine and ecstasy. Additionally, the RC/RA administers an assessment of risky opioid use via Timeline Follow-Back method at 90 and 180 days. This assessment will measure days of risky opioid use should the monthly assessments yield low response rates. The Psychiatric Diagnostic Screening Questionnaire (PDSQ) [41], is a brief self-administered instrument to assess DSM-IV alcohol and drug use disorders. The PDSQ was chosen as the only validated self-administered tool for DUD and AUD and was validated in more than 3000 patients in both medical and mental health outpatient settings [39, 42, 43]. Because it does not distinguish which substance meets criteria for DUD, the CIDI is administered by the RA/RC for OUD at baseline, 6, and 12 months. Overdose risk is assessed by the Overdose Risk Behavior Questionnaire, [15, 44–46] and past month overdose experience is assessed through a single question used in prior research.[47].

For non-substance use measures, see Table 2.

Primary care provider outcomes focused on improving guideline-concordant practices with respect to patients with risky opioid use, including prescribing lower doses of opioids, avoiding concomitant benzodiazepines, increased monitoring and higher visit frequency. Provider-level outcomes are measured through chart abstraction in the Electronic Health Record, including opioid prescriptions (< 50 Morphine Milligram Equivalent (MME), 50–90 MME and =  > 90 MME), benzodiazepine prescriptions, naloxone prescriptions, number of urine drug screens ordered and completed, new diagnoses of OUD, and number of scheduled primary care visits per patient participant.

### Other outcomes

Other measures will be collected to assess exploratory outcomes, to characterize domains from our conceptual model, and may be used to adjust models of primary and secondary outcomes. See Table 2 for details.

Qualitative interviews of up to 30 participants in the intervention condition after 3 months will explore how they engaged with the different components of the intervention (NCM, Health Coach, PCP brief advice). Qualitative interviews are guided by the social ecological framework [48] to examine barriers and facilitators to reducing opioid use and intervention engagement related to patient characteristics, social networks, the community, and the health care system. Interviews will be audio-recorded, transcribed, and analyzed using thematic analysis techniques [49].

Study assessments are estimated to require one to two hours at baseline and 3, 6,9 and 12 months. The Monthly health living survey takes participants approximately 15 min.

### Fidelity measures

To evaluate fidelity to the STOP intervention model, each person (PCP, NCM, Health Coach) completes measures about interventions delivered. For the PCP, this includes a checklist reporting what elements of the brief advice they delivered, and the time required. The NCMs document delivery of the intervention including number of interactions, type of interactions (e.g., text, phone, in-person visit), and the duration and content of those interactions. Health coaches complete and session content checklist at the conclusion of each session. In addition all coaching calls are recorded (when patient participant gives permission) and coded by a trained, independent rater for use of motivational interviewing and cognitive behavioral therapy, using the Motivational Interviewing Treatment Integrity (MITI) Scale 4.1 [50, 51] and following the strategy of Haddock and colleagues to integrate elements of CBT into the fidelity monitoring [51]. All sessions are recorded for the first three months of the study to verify that Health Coaches are achieving consistent delivery of the intervention. Afterwards, a random 10% of the sessions are evaluated for adherence to the intervention.

At the end of study participation, the last survey that all patient participants complete is an assessment of what they think the study was targeting (opioids, diet, exercise, etc.).

### Analysis

A mixed effects negative binomial model with a log link will be fit to estimate the difference in mean number of days of risky opioid use in the past 6 months between treatment and control groups. The model will include random PCP intercepts to account for within-PCP correlation of participant response values, fixed site effects to account for within-site correlation of responses, a fixed treatment effect, and the baseline value of the response variable (days of use within 30 days prior to the baseline assessment timepoint), which may improve precision. Letting Y_jk_ denote the response variable of a participant in site j with PCP k, *treatment* denote a binary treatment indicator variable, and *baseline* denote the baseline value of the outcome variable, the formula for the log-transformed expectation of Y_jk_ is below:

$${\text{log}}\left[ {{\text{E}}\left( {{\text{Y}}_{{{\text{jk}}}} } \right)} \right]{\mkern 1mu} = {\mkern 1mu} \beta _{0} + {\mkern 1mu} \beta _{1} \cdots {\text{treatment }} + {\mkern 1mu} \beta _{2} \cdots {\text{baseline }} + {\mkern 1mu} \alpha _{{\text{j}}} + {\mkern 1mu} \gamma _{{\text{k}}}$$

where α_j_ is the fixed effect for site j and γ_k_ is the random intercept for PCP k. We assume the PCP random intercepts are normally distributed with mean zero and standard deviation σ_γ_. The negative binomial model allows for potential overdispersion of the response variable and is more flexible than a Poisson distribution which would require the mean to be equal to the variance. If 2 or more PCPs are randomized as a single cluster because they practice as a team, they will be analyzed as a single cluster. The primary hypothesis will be evaluated by testing whether the treatment effect, β_1_, is different from zero. This is equivalent to testing whether the control mean is different from the intervention mean.

The primary analysis will be performed on the Intention-to-Treat (ITT) principle, analyzing patient participants according to their randomization assignment regardless of potential exposure to the opposite assignment. The primary analysis will use multiple imputations to account for missingness of the primary outcome variable. The imputation model will incorporate information on days of risky use from the timeline follow back surveys. Further details of the imputation model will be specified in the Statistical Analysis Plan, which will be finalized prior to the primary analysis is performed. A secondary analysis will be performed on the Per Protocol (PP) population. This population will exclude patient participants who had appointments with PCPs in the opposite treatment arm. The primary outcome will be evaluated using a two-sided test with a type I error rate of 5%.

Secondary analyses of the primary outcome include the model used in the primary analysis adjusted for individual-level covariates which may be associated with the response.

### Analyses of secondary outcomes

A secondary analysis will fit the same model to the 180-day outcome measured at the 12-month timepoint to assess durability. Further secondary analyses will explore temporal patterns of number of days of risky opioid use. In addition, a mixed effects negative binomial model will be fit including treatment by time interactions for each timepoint. The model will include participant random intercepts to account for correlation in responses arising from multiple observations for each patient participant.

Analyses for Aim 2 secondary outcomes will be analyzed with mixed effects regression models with treatment by time interactions (analogous to those described above for the primary outcome) or generalized linear models with generalized estimating equations (GEE) to account for correlation of responses will be fit. Similar regression models will be fit for the Aim 3 objectives, but these will use provider participant rather than patient participant as the unit of observation.

### Sample size estimation/Statistical Power

Power was calculated based on simulations exploring four possible scenarios to consider different time trends of the intervention effect as well as a possible assessment impact in the control group. Assessment impact means that the regular recording of opioid use in the monthly assessments causes self-awareness which leads to behavior modifications [52, 53]. The four scenarios are depicted in Additional file 1: Appendix Figure S1. This figure shows the mean values of risky opioid use days (within the past 90 days) assumed in each arm at 0, 3, and 6 months. The mean in the control arm is expected to be between 10 and 20, and 20 was selected for simulations because it is the most conservative (i.e., gives the lowest power), assuming that the additive effect size does not change as the control group mean changes. Scenario 1 has a 6-day constant difference between groups at both time points; in scenario 2 the intervention effect wanes to a 5-day difference during months 4–6. Scenarios 3 and 4 are the same as 1 and 2, but with assessment impact operationalized by decreasing the mean by an additional 1 day per 90 days in each arm. For more detailed statistical information, see Additional file 1.

The original protocol planned to enroll 60 PCP clusters with approximately 8 patient participants per PCP cluster for a total of 480 patient participants. The results of the simulations showed power for all 4 scenarios with 60 PCP clusters and 8 patient participants per PCP cluster to be at least 95%. During the trial, patient recruitment challenges led to a re-evaluation of whether sufficient power could be maintained with a reduced sample size, given that the study had been very highly powered. Therefore, we re-ran the power simulations and showed that that the following two conditions were all four scenarios had adequately power (89–99%) for the following two sample sizes: 1) 60 PCP clusters with 5 patients per PCP cluster for a total of 300 patient participants and 2) 50 PCP clusters with 4 patients per PCP cluster for a total of 200 patient participants, with power for all 4 scenarios for both conditions in the range of 89% to 99%.

Based on these results and the observation that the study had not yet been able to identify eligible patients for 30–40% of enrolled PCPs, the study was modified to plan for enrollment of approximately 100 PCPs from a total of 5 sites, which should leave approximately 60 PCP clusters with patients enrolled into the study (noting that only 1 site practices in teams). Based on prior studies of general population prevalence of opioid misuse of 3.9–6.6% [7, 17] and estimates of misuse of 30% among patients prescribed opioids, we estimated that each PCP cluster would have approximately 6–7 eligible patients of whom 75% will be enrolled into the study, averaging 5 patient participants per enrolled PCP cluster. This equates to 300 patient participants participating in the study (60 PCP clusters with 5 patient participants per cluster). Based on this work, the target sample size was reduced to 300 participants following review by the Data and Safety Monitoring Board.

### Trial status and protocol version

The study protocol was developed prior to the COVID-19 pandemic and received IRB approval in January 2020. The pilot study, scheduled to run at one site in spring 2020 and the main study, scheduled to start in September 2020, were greatly impacted by the COVID 19 pandemic, pushing all timelines back and requiring major adjustments to many aspects of the study protocol, especially the recruitment methods. Study recruitment of PCPs began in January 2021. Patient recruitment began in March 2021. Patient recruitment will be completed April 30, 2023. This manuscript was based upon Protocol Version 7.0.

### Data safety and monitoring board

The NIDA CTN DSMB affiliated with this trial is responsible for conducting periodic reviews of accumulating safety, trial performance, and outcome data.

## Discussion

This paper describes the design and protocol of one of the first multi-site randomized controlled trials to examine interventions for adults presenting in primary care settings with subthreshold opioid use disorder [9]. It addresses an important gap in the literature: most trials addressing risky and potentially harmful opioid use have focused on either patients who already have moderate to severe opioid use disorder, or on prescribing opioids for pain. In contrast, our study focuses on individuals who have only risky opioid use behaviors and are currently experiencing few if any consequences to their health or functioning resulting from opioid use, but who nonetheless may be at risk for opioid-related harms, including to the development of moderate or severe OUD.

Little is currently known about the trajectories of individuals who have subthreshold OUD, including how likely, and within what timeframe, they may develop a more severe OUD or experience opioid-related overdose or other serious negative health impacts. OUD prevention efforts have focused on regulatory efforts and published guidelines about prescribing opioid analgesics, particularly by primary care physicians [54, 55]. There is increasing concern that curbing prescribing could be contributing to the opioid crisis when opioid tapers are not accompanied by interventions that actively engage patients and providers in preventing the development of OUD, treat underlying pain, and provide access to effective treatment and overdose prevention strategies for individuals who already have a moderate-severe OUD [56–58].

In its 2020 recommendation to screen adult primary care patients for drug use, the US Preventive Services Task Force (USPSTF) noted critical gaps in the evidence for interventions to reduce drug use, risk behaviors, and negative health consequences. In particular, evidence was noted to be lacking for treatment interventions among non-treatment seeking patients who are identified via screening.[59–61] Although it is limited specifically to patients with subthreshold OUD, our study will contribute to filling this evidence gap, and may inform future studies of primary care interventions for other types of drug use.

The STOP collaborative care model brings together intervention components that individually improved outcomes in opioid prescribing and substance use but have not previously been tested in combination or with a focus on subthreshold OUD. Collaborative care has been shown to improve patient outcomes and increase quality of care for a variety of conditions, including alcohol use disorder and depression [21, 27, 62–65]. The small number of studies that have previously examined collaborative care interventions for drug use did not show significant changes in opioid use [21, 66]. However, these trials enrolled a different treatment population, comprising individuals with moderate-severe SUD rather than subthreshold OUD. Patients with subthreshold OUD, who are the focus of our trial, have less severe symptoms and different treatment needs, requiring less emphasis on medications for OUD treatment and more focus on motivating behavior change, medical care coordination, and self-management. A feature of the STOP collaborative care intervention, which was designed to address these needs, is the addition of an off-site telephone health coach providing support and counseling using techniques from motivational interviewing and cognitive behavioral therapy. The Health Coach has the potential to engage patients in setting goals for behavior change, while the ongoing support of the nurse care manager and PCP can enable them to access treatment and other resources that are needed to execute and sustain reductions in risky opioid use.

The intervention is designed to be integrated into regular primary care practice in clinical settings with collaborative care teams, which are increasingly common in primary care practices, but generally focus on mental health rather than substance use. The telephone health coach is also scalable in that a single health coach can service multiple sites. A strength of the STOP intervention model is that it avoids over-burdening the PCP and makes use of existing resources. Apart from the brief advice, the case management and health coaching do not require direct PCP involvement and can offload some of the work that generally falls to the PCP. Many primary care practices have allied health resources to assist with chronic care management, (for example, diabetes educators) that could be trained in providing case management and support for these high-risk patients. Telephone health coaching is commonly available through health insurance plans or large health systems. Health insurance companies could contract with national experts who do short term coaching telephonically who also collaborate with the affiliated clinical nursing resources. Should our trial have positive findings, there is thus potential for adapting and integrating the STOP intervention into existing team-based primary care models.

A notable aspect of the study design is the use of monthly self-administered assessments of opioid misuse, which can avoid recall bias and offers multiple opportunities for data collection. The strategy of inviting patient participants by text message or email to complete brief self-administered assessments that can be completed on their phone or computer has similarities to ecological momentary assessment (EMA) approaches, which often achieve high rates of completion. One of the longest duration EMA studies in the literature was an alcohol study that used weekly assessments collected by text message for 25 weeks and found that 82% of participants completed all 25 of the assessments [67]. In prior systematic reviews, allowing surveys to be completed using e-mail and text messages typically results in higher response rates [68, 69]. By easing barriers to their completion we thus anticipate having a high participation rate. While there is biological verification with a urine drug screen, this verification captures a short window of substance use, and is used as an exploratory measure.

## Limitations

Limitations include the challenges in identifying individuals meeting the eligibility criteria and in quantifying low level risky opioid use. Risky opioid use and OUD are not well captured in electronic health records ,[70] and so we rely on screening delivered as part of the research to identify eligible participants. Changes in patient receipt of prescription opioids may be limited as the intervention takes place in primary care settings, but the controlled substance may be prescribed elsewhere. The use of prescribed opioids, including those from outside providers, is only measured if patients report using them nonmedically.

The primary outcome of days of risky opioid use for this trial comes from validated tools but itself has not been validated. Existing opioid-specific screening tools can predict likelihood of opioid misuse but do not quantify or identify risky opioid use. ^16^ To overcome this limitation, the study team used items from the validated COMM assessment that relate to specific opioid consumption behaviors (taking more than prescribed or taking medication that belonged to/was borrowed from someone else). These measures were then quantified for the primary outcome. However, these measures still require the participant to distinguish nonmedical from medically indicated use to accurately respond to these questions. For example, if a patient has an opioid prescribed to treat severe pain but then takes it to help with their sleep or for mood elevation, they may not perceive it as taking a medication not as prescribed (since they have not exceeded the intended daily dose), even though this would be considered nonmedical use of their medication. Furthermore, the stigma of nonmedical use and fear of having their opioid medications discontinued may reduce reporting of misuse behaviors [71–73]. A key feature of addiction is an element of impaired self-awareness, including cognitive distortions around potential misuse of substances.[74, 75] Therefore, the study may not have the sensitivity and specific for detecting and confirming risky opioid use based on the underlying need for self-identification.

The study design relies on frequent self-reported assessments of substance use to assess the primary outcome. Assessment itself has been observed to motivate change in trials of early intervention approaches for risky alcohol use, [52, 53] and this could occur in our study population. Our RCT design minimizes the possibility of this as the control condition (EUC) receives identical assessments as the intervention group, the participants are blinded to the focus on opioids by including questions on other health behaviors, and planned statistical analyses account for assessment reactivity. Furthermore, we rely on confidential self-administered assessments to minimize social desirability bias in reporting.

## Conclusion

The Subthreshold Opioid Use Disorder Prevention Trial (STOP) is a cluster randomized trial that draws on successful interventions combining nurse care management and telephone health coaching with primary care to intervene with patients experiencing subthreshold OUD. Given the growing interest in interventions for managing patients with risky opioid use, and the need for primary care-based interventions, this study could offer a blueprint for a feasible and effective approach in this population.

### Supplementary Information

**Additional file 1: Table S1.** Mapping of the STOP Intervention Components to the Social Ecological Framework’s Four Levels of Influence.

## Data Availability

This study will comply with the NIH Data Sharing Policy and Implementation Guidance (https://grants.nih.gov/grants/policy/data_sharing/data_sharing_guidance.htm) and (for HEAL-funded studies) the HEAL Public Access and Data Sharing Policy (https://www.nih.gov/research-training/medical-research-initiatives/heal-initiative/research/heal-public-access-data-sharing-policy). Primary data for this study will be available to the public in the NIDA data repository, per NIDA CTN policy. For more details on data sharing please visit https://datashare.nida.nih.gov/.

## Abbreviations

BPI: Brief Pain Inventory
CBT: Cognitive Behavioral Therapy
CCM: Chronic Care Model
COMM: Current Opioid Misuse Measure
CIDI: Composite International Diagnostic Interview
EUC: Enhanced Usual Care
HEAL: Helping to End Addiction Long-term
MI: Motivational Interviewing
MITI: Motivational Interviewing Treatment Integrity
MME: Morphine Milligram Equivalent
MOUD: Medications for Opioid Use Disorder
NCM: Nurse Care Manager
OUD: Opioid Use Disorder
PCP: Primary Care Provider
PDSQ: Psychiatric Diagnostic Screening Questionnaire
PHQ: Patient Health Questionnaire
PROMIS: Patient-Reported Outcomes Measurement Information System
PSS: Patient Safety Screener
QUIT: Quit Using Drugs Intervention Trial
RC: Research Coordinator
RA: Research Assistant
RCT: Randomized Controlled Trial
SBIRT: Screening, Brief Intervention and Referral to Treatment
STOP: Sub-Threshold Opioid Use Disorder Prevention
TAPS: Tobacco, Alcohol, Prescription Medication, and other Substance Screening
TEACH: Teaching Effective Analgesia in Clinics for HIV
TLFB: Timeline Follow Back
TOPCARE: Transforming Opioid Prescribing in Primary Care
