# Supplementary material for: Subthreshold opioid use disorder prevention (STOP) trial: a cluster randomized clinical trial: study design and methods
Source: Addict Sci Clin Pract. 2023 Nov 18;18:70. doi: 10.1186/s13722-023-00424-8 (PMC10657560; doi:10.1186/s13722-023-00424-8)
Supplement: Supplementary file 1 — Additional file 1: Table S1. Mapping of the STOP Intervention Components to the Social Ecological Framework’s Four Levels of Influence. [file 13722_2023_424_MOESM1_ESM.docx]

Appendix Table 1: Mapping of the STOP Intervention Components to the Social Ecological Framework’s Four Levels of Influence

| **Levels of Influence** | **STOP Intervention Components** |
| --- | --- |
| 1. Individual | - Brief advice from PCP enhances knowledge about the health risks of opioids and increases motivation to reduce opioid misuse. - Video Doctor reinforces PCP message. - Telephone health coach provides counseling that increases motivation, self-efficacy, and skills to reduce opioid misuse. Stepped-up health coaching sessions utilize MI/CBT to target knowledge, motivation, attitudes, beliefs, and behavioral skills for treatment refractory patients, with specific emphasis on reducing opioid misuse and related risk behaviors, managing chronic pain, and/or preventing substance-related relapse. - Nurse Care Manager provides individualized patient education (including overdose prevention, risk reduction, and self-management skills), assessment, and ongoing monitoring. |
| 1. Interpersonal | - Telephone health coach educates patient participants on how to talk with peers about opioid use and overdose prevention (including normative behavior); teaches assertive communication with family and peers and ways to maintain their own appropriate opioid use despite others’ misuse. - Nurse care manager provides Overdose Education and Naloxone Distribution (OEND), including education to members of patient participants’ social networks, and coaches patient participants on communicating their needs effectively to their clinical care team. |
| 1. Organizational (Clinic level) | - Telephone health coach suggests clinic resources and proactively links patients to the Nurse Care Manager for referrals when patient participants spontaneously raise barriers to reducing opioid misuse (e.g., physical or mental health problems). - Nurse Care Manager evaluates patients for OUD and opioid-related risk behaviors and provides ongoing monitoring that is communicated back to the clinical team; makes referrals to specialty care services patient participants may require (e.g., mental health or substance use disorder treatment), and links patient participants who develop OUD to treatment (office-based buprenorphine or referral to opioid treatment program). |
| 1. Community | - Telephone health coach recommends community and/or online resources and proactively links patient participants to the Nurse Care Manager and/or clinic social workers for assistance when patient participants spontaneously raise barriers to reducing opioid misuse (e.g., social determinants of health, family/friend factors, community/neighborhood factors). - Nurse Care Manager makes referrals to community services that may benefit patient participants such as physical activity programs for pain (e.g., yoga, Tai Chi), peer support for substance use (e.g., 12-step and other groups), and addiction treatment resources. |

**Appendix- PCP Brief Advice Script- Prescribed Opioids**

Please counsel your patient as follows.

1. You completed a questionnaire that included questions about your opioid use and I wonder if you had any concern about how you are using your opioid medication?
2. Can I give you some feedback based on that questionnaire?
3. I am concerned you may be using opioids in a risky way based on the score of your questionnaire. You could have problems not only now but also in the future, if you keep using in the same way.
4. This is a **very important health problem** because just like how fatty foods can lead to a disease of the heart, how you use opioids, including your opioid pain medication, can lead to a **chronic disease of the brain called addiction** that can be **long lasting** and lead to **loss of control**.
5. **Not taking your medication as prescribed** is risky because it can lead to **problems in life**, like problems with your family, job, or school, and car accidents. People can overdose without meaning to, even **when taking what was prescribed by a clinician**. This is especially true if opioids are taken **with alcohol, sedatives (benzos), or sleeping pills**.
6. It is **important** that you **take your opioid medication exactly as prescribed** to lower your risk of developing addiction and other health and social problems.
7. Are you **willing** over the **next month** to **take your medication exactly as I prescribed it?**

If Patient Tests Positive for At Risk Use or Possible Addiction to Other Substances

1. I am also concerned about your **risky use** of ___________________________________________, which can also cause health problems and puts you at risk of developing an addiction and overdose.

For ALL Patients

1. **Your telephone health coach** will call you in **2 and 4 weeks** to support you in lifestyle changes you want to make to improve your health.
2. Nurse (NAME) is very experienced and will be partnering with me in caring for you. The NURSE will focus on your whole self, including your physical and mental health. As part of your care team, Nurse (NAME) will be working with you, me, and the Telephone Health Coach.
3. Here is a **report card** of your answers to the Healthy Living Survey that led me to talk to you about changing your **________________**use behavior because of the harm it might cause you.
4. Here is an educational pamphlet about how to prevent opioid overdose, and your nurse can provide you an emergency naloxone kit and teach you how to use it.
5. We can check in about your **________________**use again at your **next visit**.

**Note to provider: Below are the results of the patient’s Healthy Living Study screener.**

TAPS Score Risk Category

In past 3 months:

0 No Use

1 Problem Use

2+ Higher Risk

|  | **TAPS Score** |
| --- | --- |
| **Tobacco** |  |
| **Alcohol** |  |
| **Marijuana** |  |
| **Stimulants (including cocaine, methamphetamine)** |  |
| **Heroin or fentanyl** |  |
| **Rx opioids** |  |
| **Sedatives (benzodiazepines)** |  |
| **Rx stimulants (amphetamines)** |  |
| **Other illegal or recreational drugs** |  |

| **Patient reported** (on COMM): | In past 30 days: |
| --- | --- |
| needed to take pain medications belonging to someone else |  |
| had to take more of your medication than prescribed |  |
| borrowed pain medication from someone else |  |

**Appendix-PCP Brief Advice Script- Illicit and Non-prescribed use**

Please counsel your patient as follows.

1. You completed a questionnaire that included questions about your opioid use and I wonder if you had any concern about how you are using opioids (like heroin or prescription opioid pain medications)?
2. Can I give you some feedback based on that questionnaire?
3. On your questionnaire, **you scored at risk for developing problems related to your use** of opioids. You could have problems not only now but also in the future, if you keep using in the same way.
4. This is a **very important health problem** because just like how fatty foods can lead to a disease of the heart, how you use opioids can lead to a **chronic disease of the brain called addiction** that can be **long lasting** and lead to **loss of control** where you **can’t stop** using the opioid. Your brain and body can become addicted to **opioids after just two weeks of using opioids** every day.
5. Opioid use is risky because it can lead to **problems in life**, like problems with your family, job, or school, and car accidents. People can overdose without meaning to, even with **low levels of use**, especially if opioids are taken **with alcohol, sedatives (benzos), or sleeping pills**. Injecting drugs increases risk for HIV, Hepatitis B and Hepatitis C.
6. It is **important** that you **quit or reduce** your use of opioids**,** since you **may still have the power to do so now** before you develop the chronic brain disease called addiction and other health and social problems.
7. Are you **willing** in the **next month** to **quit or reduce** your opioid **use**?

If Patient Tests Positive for At Risk Use or Possible Addiction to Other Substances

1. I am also concerned about your **risky use** of ___________________________________________, which can also cause health problems and puts you at risk of developing an addiction and overdose.

For ALL Patients

1. **Your telephone health coach** will call you in **2 and 4 weeks** to support you in lifestyle changes you want to make to improve your health.
2. Nurse (NAME) is very experienced and will be partnering with me in caring for you. The NURSE will focus on your whole self, including your physical and mental health. As part of your care team, Nurse (NAME) will be working with you, me, and the Telephone Health Coach.
3. Here is a **report card** of your answers to the Healthy Living Survey that led me to talk to you about changing your **________________**use behavior because of the harm it might cause you.
4. Here is an educational pamphlet about how to prevent opioid overdose, and your nurse can provide you an emergency naloxone kit and teach you how to use it.
5. We can check in about your **________________**use again at your **next visit**.

**Note to provider: Below are the results of the patient’s Healthy Living Study screener.**

TAPS Score Risk Category

In past 3 months:

0 No Use

1 Problem Use

2+ Higher Risk

|  | **TAPS Score** |
| --- | --- |
| **Tobacco** |  |
| **Alcohol** |  |
| **Marijuana** |  |
| **Stimulants (including cocaine, methamphetamine)** |  |
| **Heroin or fentanyl** |  |
| **Rx opioids** |  |
| **Sedatives (benzodiazepines)** |  |
| **Rx stimulants (amphetamines)** |  |
| **Other illegal or recreational drugs** |  |

| **Patient reported** (on COMM): | In past 30 days: |
| --- | --- |
| needed to take pain medications belonging to someone else |  |
| had to take more of your medication than prescribed |  |
| borrowed pain medication from someone else |  |

Appendix—Copy of Monthly Healthy Living Survey

1. During these 30 days (dates of assessment), how often did you eat fast food meals?

Daily or almost daily, Weekly, Once or twice, never, don’t know/not sure

1. During these 30 days (dates of assessment), how often did you drink sugar sweetened beverages?

Daily or almost daily, Weekly, Once or twice, never, don’t know/not sure

1. During these 30 days (dates of assessment), how often did you exercise for at least 20 minutes?

Daily or almost daily, Weekly, Once or twice, never, don’t know/not sure

1. Do you currently have a prescription for an opioid pain medication?

*These includes medications like morphine (MS-Contin), oxycodone (OxyContin, Percocet), hydrocodone (Vicodin, Norco), methadone, codeine, tramadol (Ultram) and similar opioid pain medications that require a prescription from a medical provider.*

No/Yes

1. During these 30 days (dates of assessment), on how many days did you smoke at least one cigarette (enter 0 for never)? ____
2. During these 30 DAYS (See “Assessment Period” at the top of this page), on how many days did you have 4 or more drinks containing alcohol in a day?

(Enter “0” for never)

*Consider a “drink” to be a can or bottle of beer (12 ounces), a glass of wine (5 ounces), or a shot of hard liquor like gin, vodka, or whiskey (1.5 ounces).* __________

*If yes to 6, answer 7; If no to 6, skip to 8*

1. During these 30 DAYS (See “Assessment Period” at the top of this page), on how many days did you have 5 or more drinks containing alcohol in a day?

(Enter “0” for never)

*Consider a “drink” to be a can or bottle of beer (12 ounces), a glass of wine (5 ounces), or a shot of hard liquor like gin, vodka, or whiskey (1.5 ounces).* __________

This section includes questions about your use of opioid pain medications.

These include medications like morphine (MS-Contin), oxycodone (OxyContin, Percocet), hydrocodone (Vicodin, Norco), methadone, codeine, tramadol (Ultram) and similar opioid pain medications that require a prescription from a medical provider.

1. During these 30 DAYS (See “Assessment Period” at the top of this page), on how many days did you need to take pain medications belonging to someone else? *(Enter “0” for never)*

*Note: This question is asking only about your use of opioid pain medications.*

___ days

1. During these 30 DAYS (See “Assessment Period” at the top of this page), on how many days did you have to take more of your medication than prescribed? *(Enter “0” for never)*

*Note: This question is asking only about your use of opioid pain medications.*

___ days

1. During these 30 DAYS (See “Assessment Period” at the top of this page), on how many days did you borrow pain medication from someone else? *(Enter “0” for never)*

*Note: This question is asking only about your use of opioid pain medications.*

___ days

1. During these 30 DAYS (See “Assessment Period” at the top of this page), on how many days did you use your pain medication for symptoms other than for pain (e.g., to help you sleep, improve your mood, or relieve stress)? *(Enter “0” for never)*

*Note: This question is asking only about your use of opioid pain medications.*

___ days

1. During these 30 DAYS (See “Assessment Period” at the top of this page), did you ever use your own prescription opioids medications more than prescribed **on the same day** that you used opioid medications that belonged to someone else?

No/Yes

*If your response is “Yes” to Q12, answer Q13.*

*If your response is “No” to Q12, skip to Q15.*

1. During these 30 DAYS (See “Assessment Period” at the top of this page), on how many days did you use your own medication more than prescribed **on the same day** that you needed opioid mediations belonging to someone else? ______ days
2. During these 30 DAYS (See “Assessment Period” at the top of this page), on how many days did you use **prescription opioid medications** that were not prescribed to you? *(Enter 0 for never)* ______ days

*These include opioid pain relievers such as OxyContin, Vicodin, Percocet, Norco, and Methadone.*

1. During these 30 DAYS (See “Assessment Period” at the top of this page), on how many days did you use **heroin or fentanyl**? *(Enter 0 for never)* ______ days

*If you indicated at least one day in Q15 AND you indicated at least one day in Q8, Q9, or Q14, answer Q16.*

*If you indicated 0 days for Q8, Q9, Q14, AND Q15, skip to Q18.*

1. During these 30 DAYS (See “Assessment Period” at the top of this page), did you ever use heroin or fentanyl **on the same day** you used prescription opioid medications more than prescribed or that were not prescribed to you (including medications that belonged to someone else)? No/Yes

*More than prescribed means more frequently than prescribed and/or at higher doses than prescribed*

*If your response is “Yes” to Q16, answer Q17. If your response is “No” to Q16, skip to Q18.*

1. During these 30 DAYS (See “Assessment Period” at the top of this page), on how many days did you use heroin or fentanyl on the same day you used prescription opioid medications more than prescribed, or that were not prescribed to you (including medications that belonged to someone else)? *(Enter “0” for never)*

*More than prescribed means more frequently than prescribed and/or at higher doses than prescribed*

1. During these 30 DAYS (See “Assessment Period” at the top of this page), on how many days did you use prescription sedative medications? (*Enter “0” for never*) _____days

*These include medications for anxiety or sleeping such as Xanax, Ativan, and Klonopin.*

*If you indicated at least one day in Q18, answer Q18a. If you indicated 0 days in Q18, skip to Q19.*

1. Were the **sedative medications** prescribed to you? No/Yes
2. On how many days did you use more than prescribed? *(Enter “0” for never*) _____days
3. During these 30 DAYS (See “Assessment Period” at the top of this page), on how many days did you use cocaine, crack, or methamphetamine? (*Enter “0” for never*) ____days
4. During these 30 DAYS (See “Assessment Period” at the top of this page), on how many days did you use cannabis (marijuana, hash, edibles, etc.)? (*Enter “0” for never*) ____days

Appendix: Listing of all Secondary Outcomes:

Patient-Level Outcomes

1. Days of risky opioid use at specified time points:
   1. In the past 30 days, measured at baseline and monthly for 12 months.
   2. In the past 90 days, assessed at 3, 9, and 12 months.
   3. In the past 180 days, assessed at 12 months.
2. Days of binge alcohol use:
   1. In the past 30 days, measured at baseline and monthly for 12 months.
   2. In the past 90 days, assessed at 3, 6, 9, and 12 months.
   3. In the past 180 days, assessed at 6 and 12 months.
3. Days of benzodiazepine use:
   1. In the past 30 days, measured at baseline and monthly for 12 months.
   2. In the past 90 days, assessed at 3, 6, 9, and 12 months.
   3. In the past 180 days, assessed at 6 and 12 months.
4. Days of stimulant use (cocaine and amphetamine-type stimulants):
   1. In the past 30 days, measured at baseline and monthly for 12 months.
   2. In the past 90 days, assessed at 3, 6, 9, and 12 months.
   3. In the past 180 days, assessed at 6 and 12 months.
5. Days of marijuana use:
   1. In the past 30 days, measured at baseline and monthly.
   2. In the past 90 days, assessed at 3, 6, 9, and 12 months.
   3. In the past 180 days, assessed at 6 and 12 months.
6. Days of other drug use (not including opioids, benzodiazepines, stimulants, and marijuana).
   1. In the past 30 days, assessed at 3, 6, 9, and 12 months
7. Increase in number of days of risky opioid use from baseline to follow-up at 6 and 12 months:
   1. Days of opioid use in the past 30 days, measured at baseline and monthly for 12 months
   2. Days of opioid use in the past 180 days, assessed at 6 and 12 months.
8. Prescription opioid misuse behaviors, among participants receiving prescribed opioids:
   1. Days of taking prescribed opioids for symptoms other than for pain, measured at baseline and monthly for 12 months.
   2. Days of taking pain medication belonging to someone else, measured at baseline and monthly for 12 months.
   3. COMM score, assessed at screening and at 6 and 12 months.
9. Substance Use Disorder
   1. Moderate-severe Opioid Use Disorder
   2. Drug Use disorder
   3. Alcohol Use disorder
10. Overdose risk behaviors and events
    1. Overdose risk behavior and behavioral intention to reduce risk is measured at baseline and at 6 and 12 months (Overdose Risk Behavior Questionnaire)
    2. Episodes of non-fatal overdose are measured at baseline and at 6 and 12 months (Non-Fatal Overdose Questionnaire)
    3. Exploratory Objective 3. Overdose death is expected to be a rare event in this population and will be assessed from the EHR and from other administrative data kept by the health system or government entities, for participants who cannot be reached at the time of the 12-month study visit.
11. Pain symptoms and pain-related functioning
    1. Pain symptoms (severity, impact on functioning) are measured at baseline and at 3, 6, 9, and 12 months using the BPI short form (items #3-6 for pain symptoms and items #9A-9G for functioning).
12. Mental health
    1. Anxiety symptoms are measured at baseline and 6 and 12 months (PROMIS short form)
    2. Depression symptoms and suicidality are measured at baseline and at 6 and 12 months (PHQ-8 and PSS)
    3. Sleep quality is measured at baseline and at 6 and 12 months (PROMIS Sleep 4a)
13. Health-related quality of life and acute health care utilization
    1. Health-related quality of life is measured at baseline and at 6 and 12 months (SF-12)
    2. ED and hospital utilization are measured using patient participant self-report of acute care events (ED visits, hospitalizations for medical reasons, hospitalizations for detoxification), collected at baseline and at 6 and 12 months.

PCP-level outcomes

1. Prescriptions for opioids: number of patient participants receiving prescriptions for high-dose opioids (>90 MME); moderate-dose opioids (50-90 MME); and any opioids: number of prescriptions; daily prescribed dose; and total number of days prescribed.
2. Prescriptions for benzodiazepines: number of patient participants receiving benzodiazepine prescriptions and number receiving both chronic opioid and benzodiazepine prescriptions: number of prescriptions; daily prescribed dose, and total number of days prescribed
3. Prescriptions for naloxone: number of patient participants receiving at least 1 prescription
4. Urine Drug Screens: number ordered and completed for each patient participant
5. Diagnosis of OUD: number of patient participants receiving a new diagnosis of OUD during the study period.
6. Primary care visits: number of scheduled visits per patient participant

Other Outcome Measures

1. Patient participants’ self-assessment of readiness and confidence to change, rated on a 10-point scale.
2. Social support assessed using the PROMIS instrumental and emotional health short forms.
3. Patient engagement in primary care (Exploratory Objective 1): Number and frequency of kept appointments and missed appointments for primary care visits.
4. Addiction treatment and harm reduction program utilization (Exploratory Objective 4): Self-reported number of weeks of addiction treatment or harm reduction program services, and self-reported number of weeks receiving MOUD is assessed at baseline and at 6 and 12 months. Prescriptions for MOUD received in the primary care clinic are additionally assessed from the EHR from at 12 months.
5. PCP knowledge and attitudes regarding substance use, subthreshold OUD, and opioid management, assessed at baseline and at the end of the intervention period.
6. TLFB (Timeline follow back) measure of substance use in the past 90 days (Exploratory Objective 5): A 90-day TLFB administered at the 3- and 6-month quarterly assessments, captures days of risky opioid use, days of binge alcohol use, and other drug use including benzodiazepines, cocaine, stimulants, marijuana, and other drugs. For any prescription opioids, benzodiazepines, and amphetamine-type stimulants, the TLFB measures non-medical use.
7. Patient participant-completed assessments measures PCP counseling on opioid use (Exploratory Objective 6). For patient participants who have a PCP encounter integrated with the baseline research visit, counseling is measured with the baseline exit survey.

Appendix Figure 1

Mean Values in Treatment and Control Groups for Four Different Simulation Scenarios


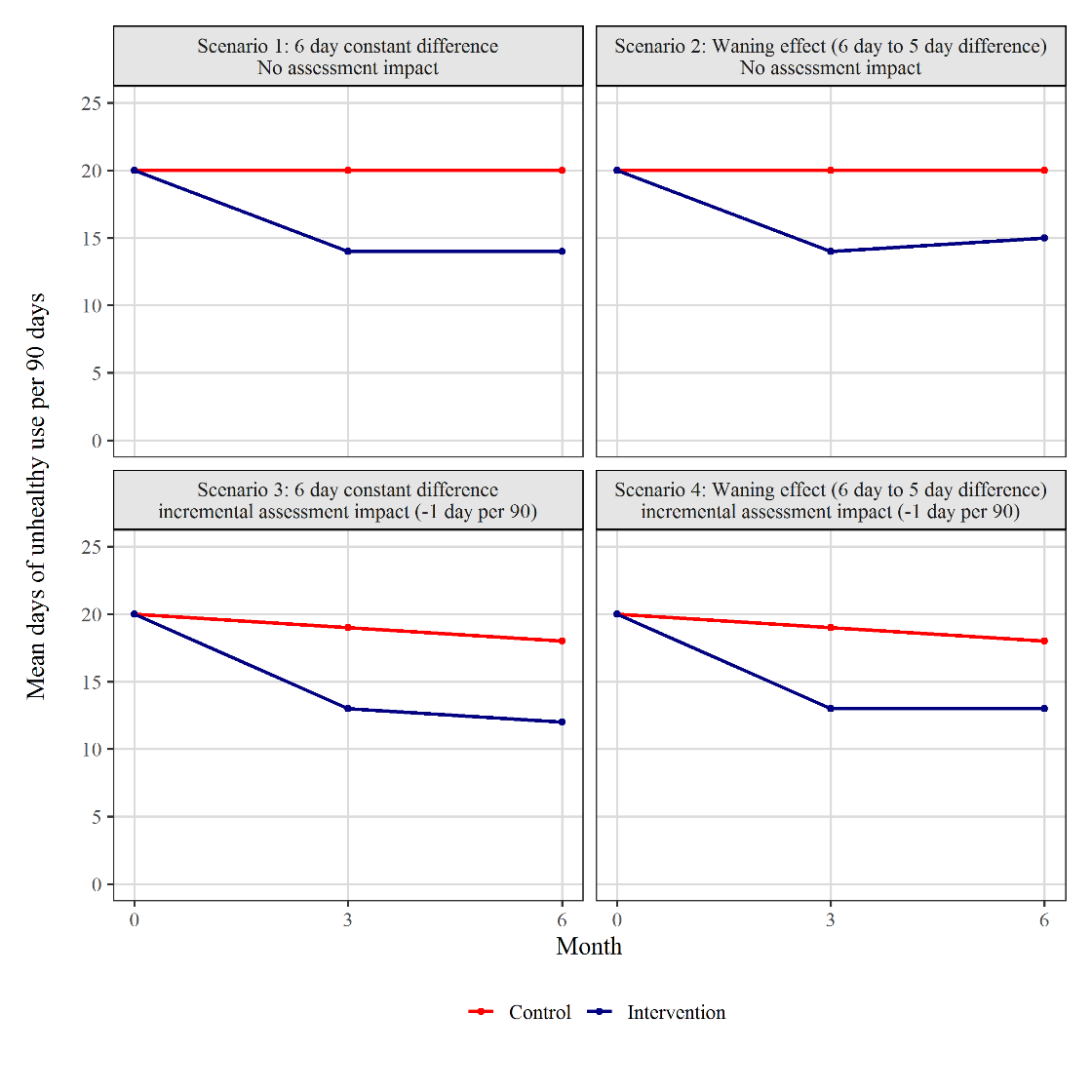


Appendix Table 2. Power estimates based on 1000 simulations for the scenarios depicted in Figure 1. This table is from the original sample size (480) including 60 PCPs with 8 patient participants per PCP.

| Scenario | Group means  (3-mo, 6-mo) | | Outcome measure and  corresponding power | |
| --- | --- | --- | --- | --- |
|  | Control | Treatment | 90-day | 180-day |
| 1: Constant effect, no assessment impact | 20, 20 | 14, 14 | 0.96 | 0.98 |
| 2: Waning effect, no assessment impact | 20, 20 | 14, 15 | 0.86 | 0.95 |
| 3: Constant effect, assessment impact | 19, 18 | 13, 12 | 0.99 | 0.99 |
| 4: Waning effect, assessment impact | 19, 18 | 13, 13 | 0.93 | 0.97 |

### Appendix Table 3: Anticipated Patient Participant Screening and Enrollment for each of the 60 PCPs Having Patients in the Study

|  | PCP Panel Size ~ 1100 patients | |
| --- | --- | --- |
|  | Prescribed Opioids  (N=8) | General Patient Population  (N=1100) |
| Number Screened (42% of panel) | 5 | 460 |
| Percent Eligible | 30% | 1% |
| Number Eligible | 1-2 | 5 |
| Agree to Enroll  (75% of eligible) | 1 | 4 |
| Anticipated Enrollees | 1 + 4 = 5 | |

Appendix- Statistical Power Calculation Details

Power was estimated by simulation as follows. Numbers of days of risky opioid use for each patient participant are drawn from binomial distributions for the two 90-day time intervals. The size parameter of the binomial distribution is 90. The probability parameter is the specified mean number of days divided by 90, but also includes a PCP intercept (randomly drawn from a normal distribution with mean zero and variance 0.15) and an individual intercept (randomly drawn from a normal distribution with mean zero and variance 0.25). The PCP intercepts induce within-PCP correlation of simulated responses. The individual intercepts create overdispersion to reflect individual variability more realistically. The variance parameters were selected to be conservative and to generate data with most values less than 30 and few values over 45 (per 90 days). The PCP variance parameter describes how much the PCP-specific mean values tend to vary from each other (which reflects the Intra Class Correlation (ICC)). With the specified parameters, the middle 50% of the PCP-specific means in the control arm lie between 17 and 25. The true within-PCP correlation is not known for this outcome, but this is expected to be an upper limit on the expected variability of the PCP-specific means so should provide a conservative power estimate. For each simulated data set, a negative binomial model was fit with random PCP intercepts and a fixed treatment effect. One thousand iterations were performed per scenario. Power was estimated as the proportion of simulated data sets with significant (p≤0.05) treatment effect estimates based on a two-sided test.
